# Supplementary figures and images for: Necroptosis in pancreatic cancer promotes cancer cell migration and invasion by release of CXCL5
Source: PLoS One. 2020 Jan 30;15(1):e0228015. doi: 10.1371/journal.pone.0228015 (PMC6991976; doi:10.1371/journal.pone.0228015)

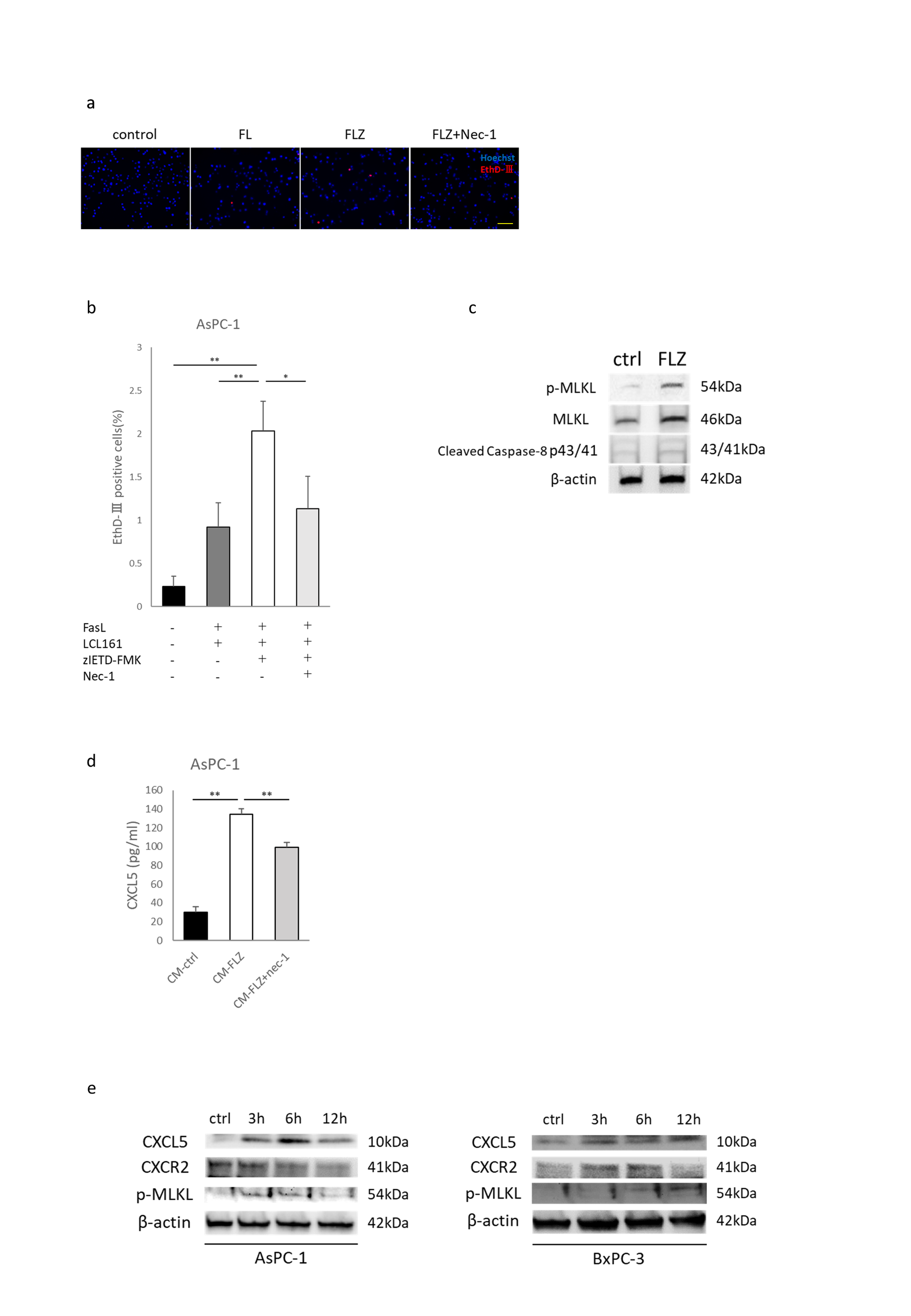

Supplement: S1 Fig — FLZ: Fas ligand, LCL161 (Smac mimetic), zIETD-FMK. (a) Fluorescent images of AsPC-1 cells treated with combination of recombinant human FAS ligand, LCL161, zIETD-FMK and nec-1. EthD-III staining shows dead cells. Scale bars = 100 μm. (b) Frequency of dead cells treated with each agent for 12 hours. (c) Western blot analysis of p-MLKL, MLKL and cleaved caspase-8. AsPC-1 cells treated with FLZ for 4 hours. (d) Concentration of CXCL5 in CM-ctrl, CM-FLZ and CM-FLZ+nec-1 measured by ELISA. (e) Western blot analysis of CXCL5 and CXCR2 expression in TSZ-treated AsPC-1 and BxPC-3 cells over time. Graphs show mean ± SE. *P < 0.05; **P<0.01. (TIF) [file pone.0228015.s001.tif]

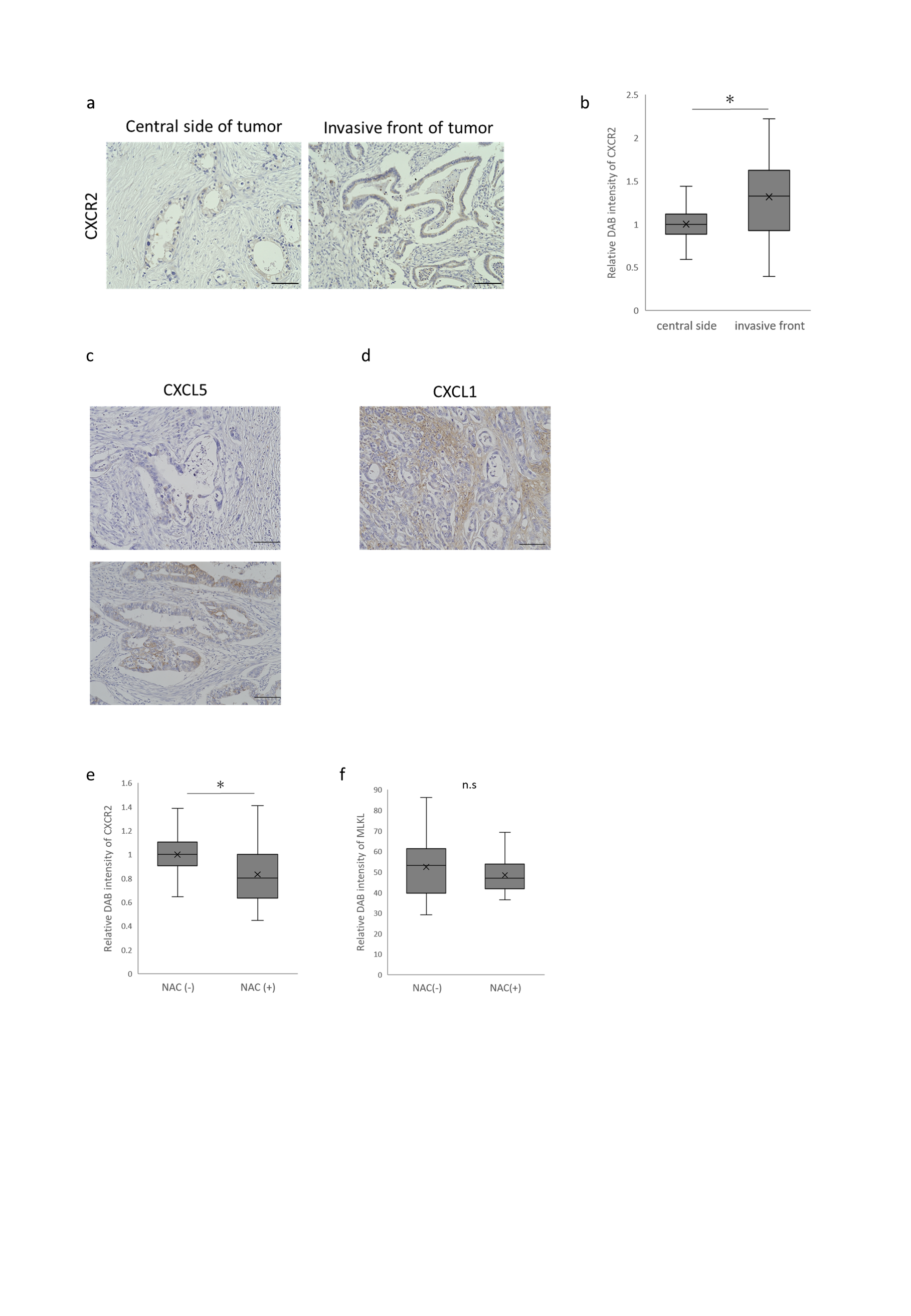

Supplement: S2 Fig — (a) Representative images of CXCR2 expression at the invasive front and center of the tumor (scale bars = 100 μm). (b) Comparison of the CXCR2 staining intensity in pancreatic cancer cells at the invasive front and center of the tumor. Five fields of view at ×200 magnification per patient were analyzed in 21 patients. (c) Representative images of CXCL5 immunohistochemistry in human PC tissues. (d) Representative image of CXCL1 immunohistochemistry in human PC. (e, f) Comparison of the CXCR2 and MLKL staining intensity at the tumor invasive front in patients who received (n = 5) or did not receive (n = 16) preoperative chemotherapy. Five fields of view at ×200 magnification per patient were analyzed. *P<0.01. (TIF) [file pone.0228015.s002.tif]
