# Supplementary material for: Necroptosis in pancreatic cancer promotes cancer cell migration and invasion by release of CXCL5
Source: PLoS One. 2020 Jan 30;15(1):e0228015. doi: 10.1371/journal.pone.0228015 (PMC6991976; doi:10.1371/journal.pone.0228015)

Fig1 d

RIP3

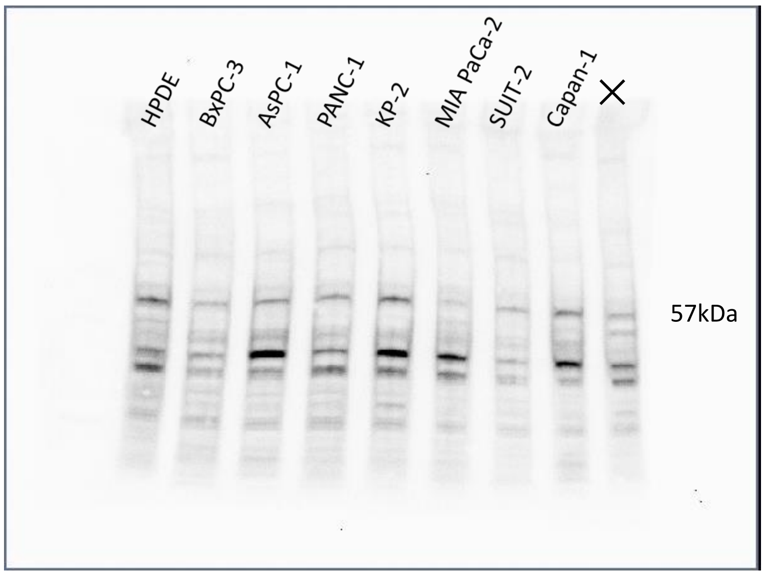

MLKL

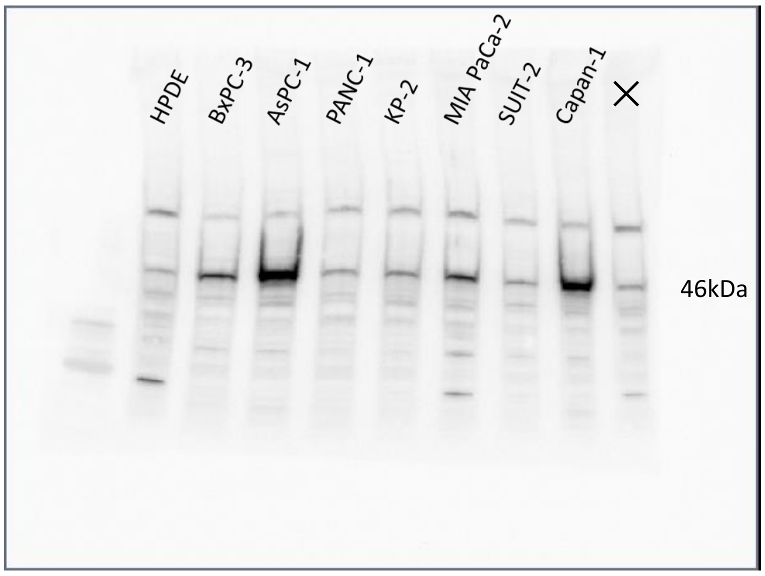

β-actin

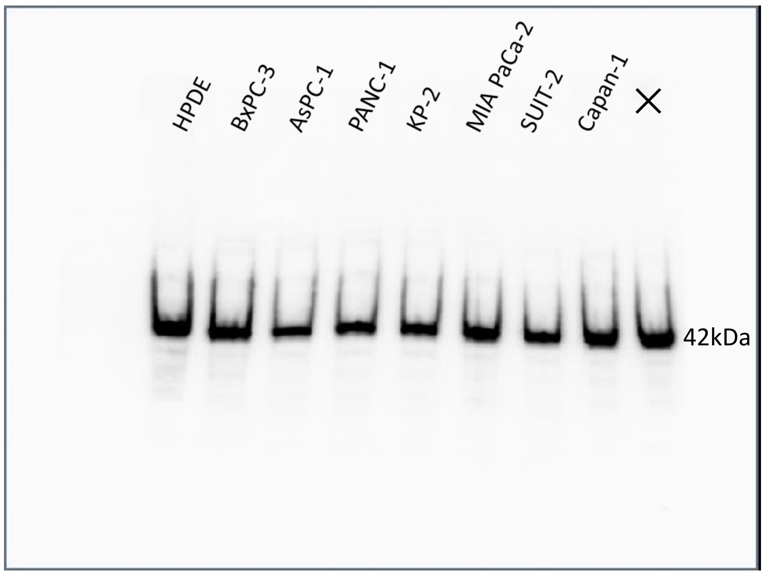

Fig2 d

p-MLKL

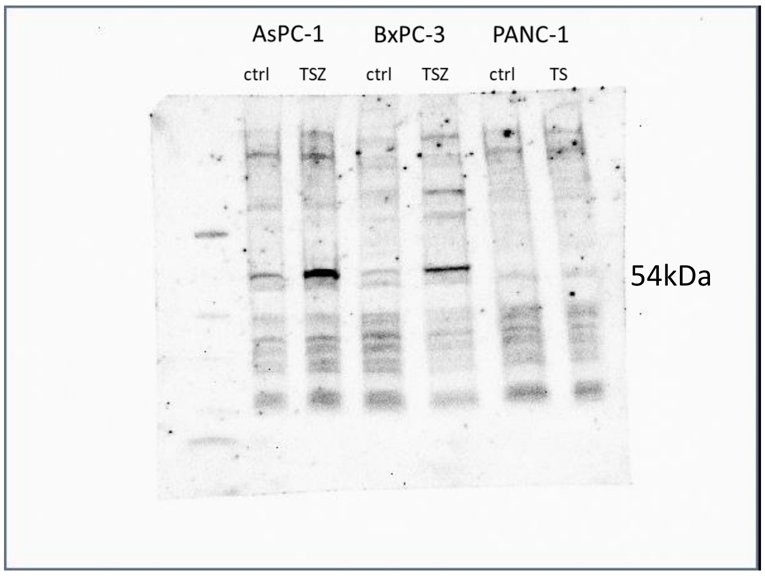

β-actin

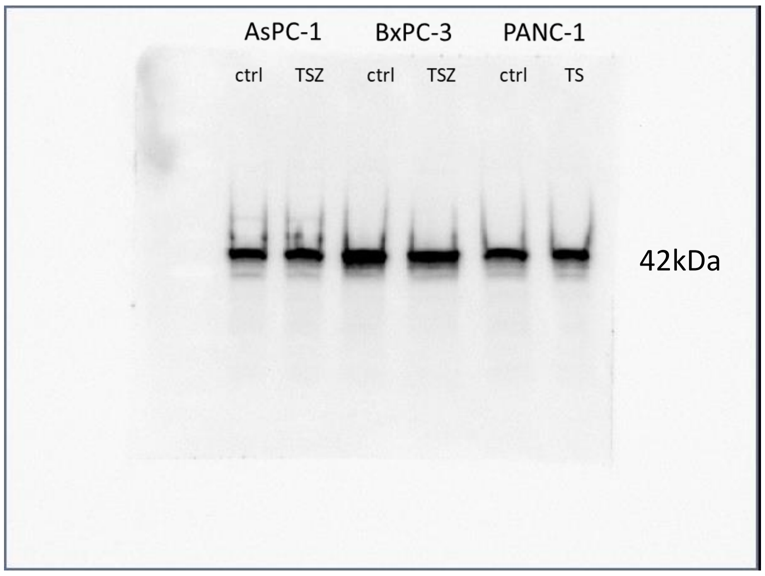

MLKL

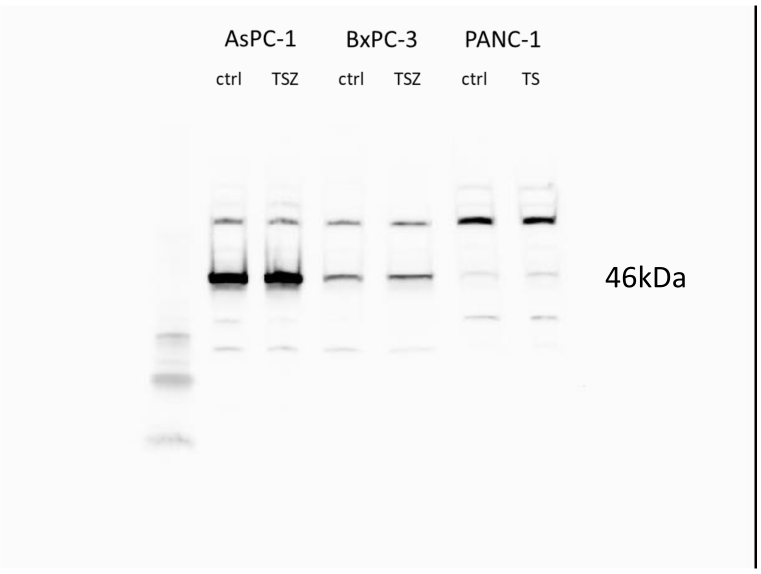

Caspase-8

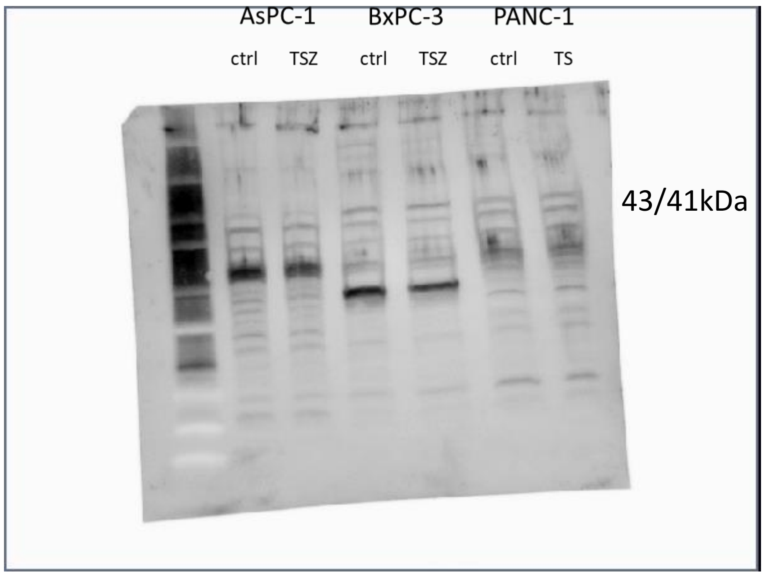

Fig4 g

CXCR2

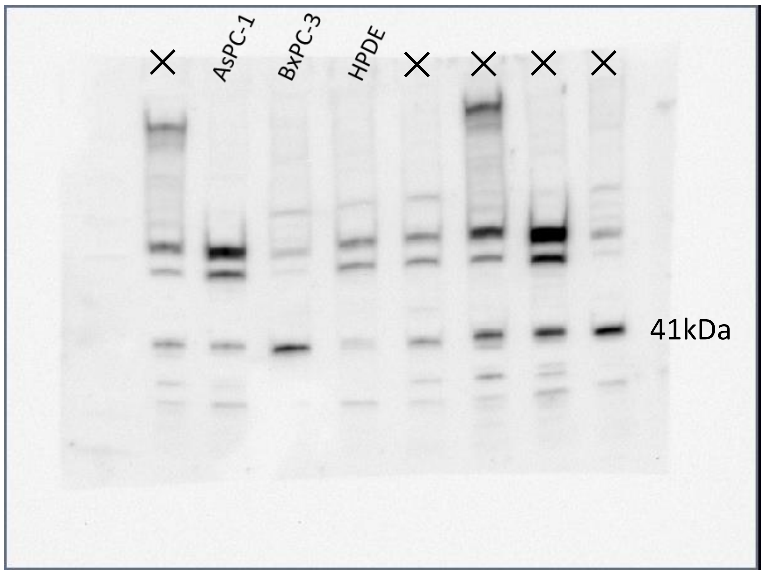

β-actin

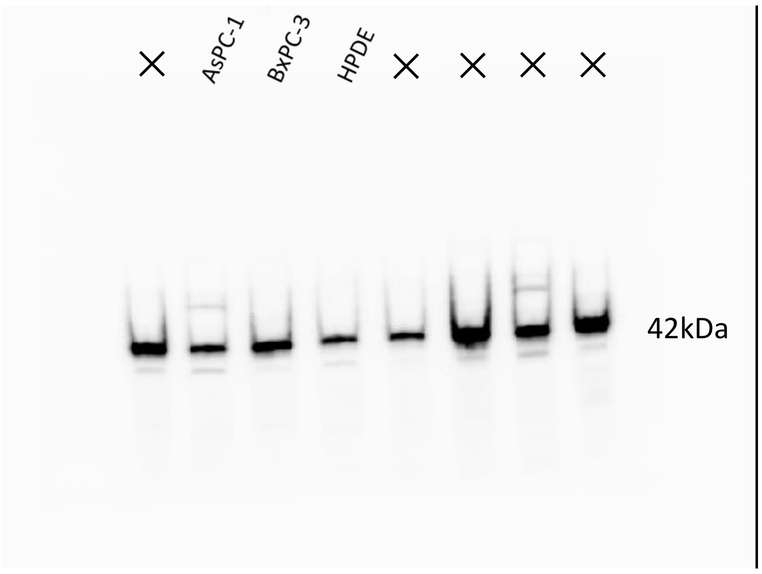

Fig5 e

CXCR2

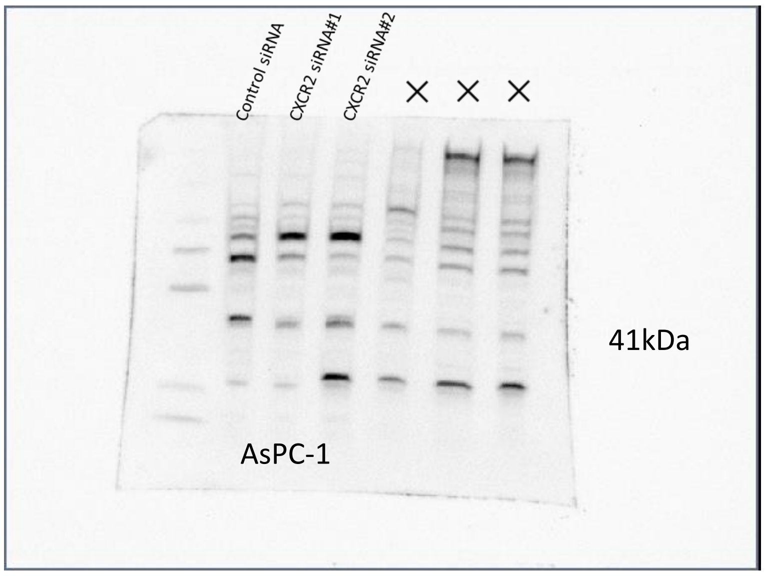

CXCR2

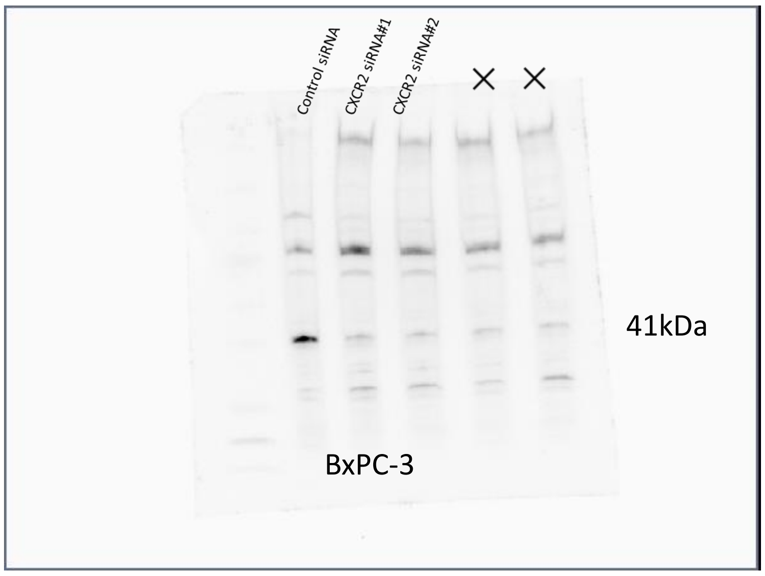

β-actin

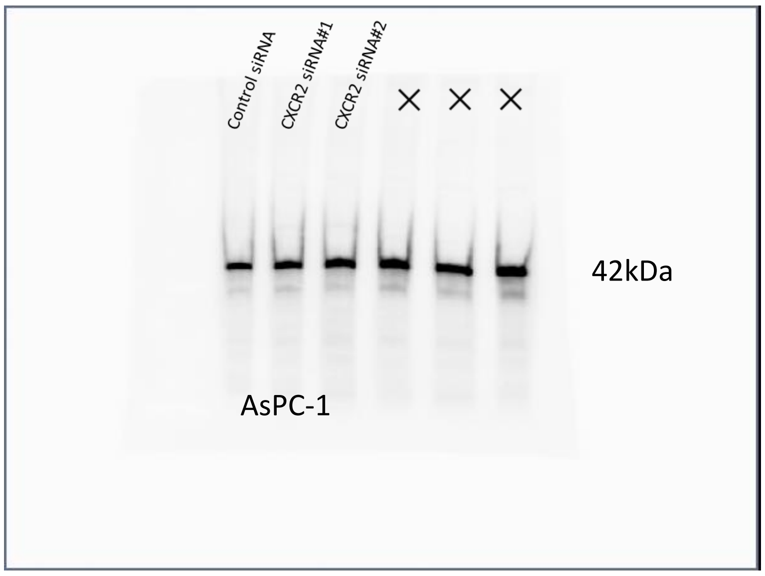

β-actin

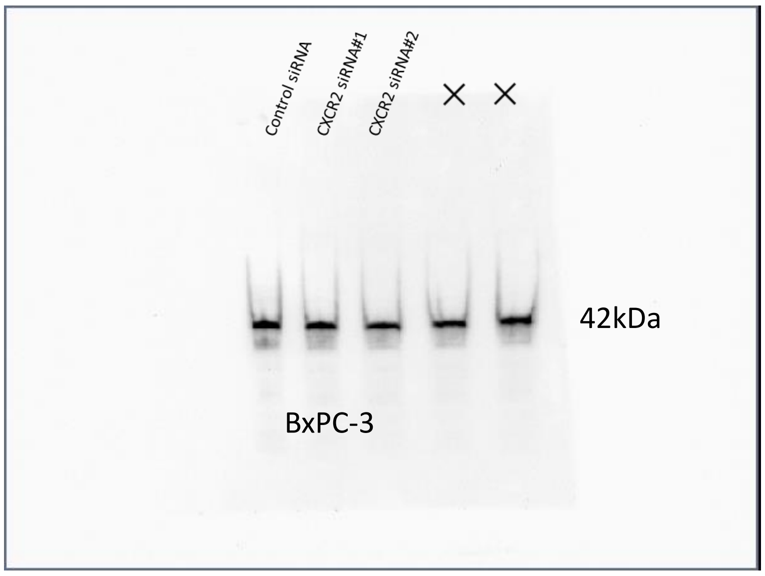

Fig7 d

CXCR2

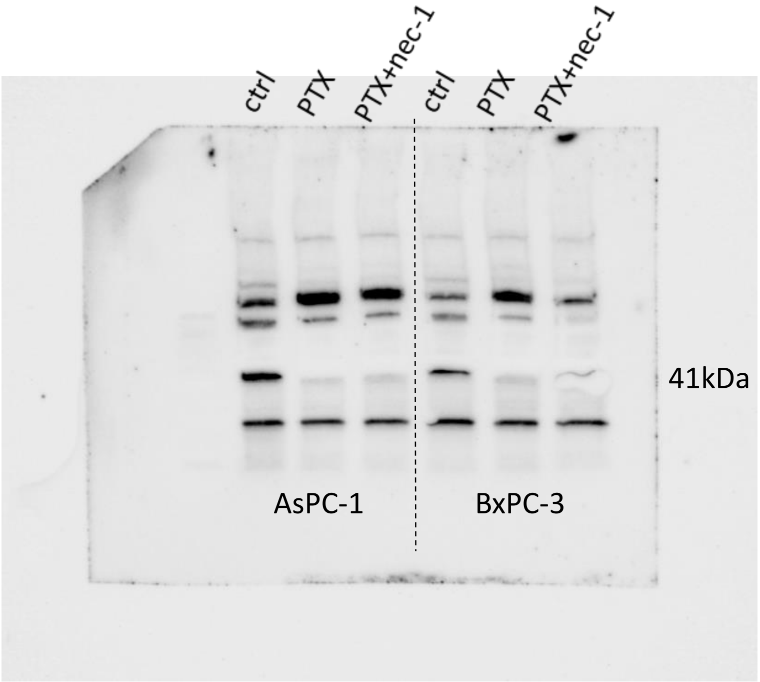

p-MLKL

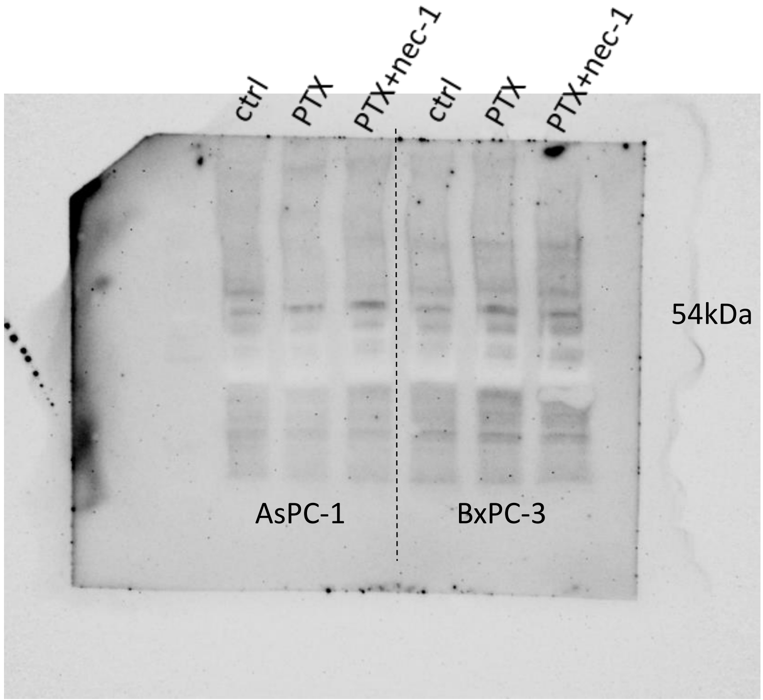

Caspase-8

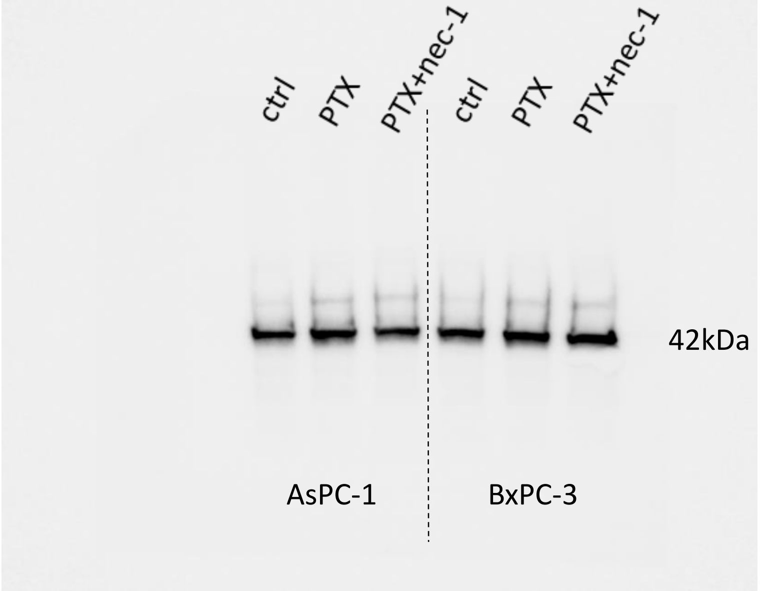

S1 Fig1c

p-MLKL

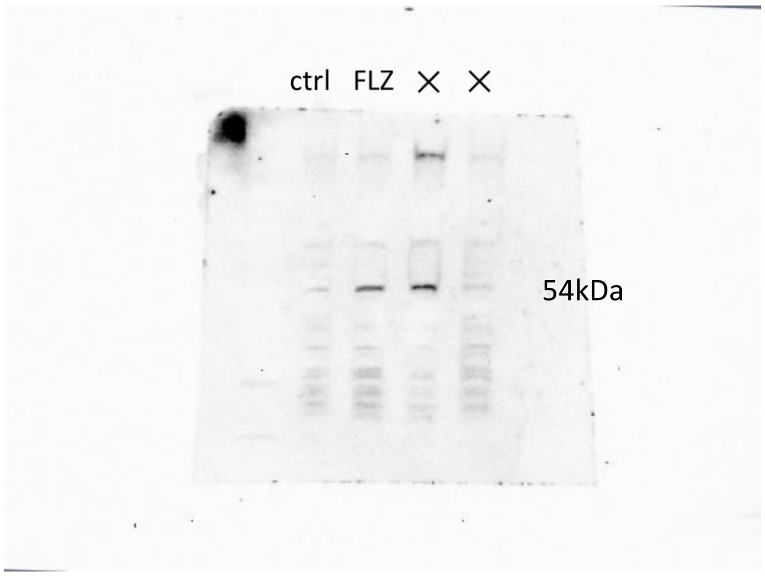

β-actin

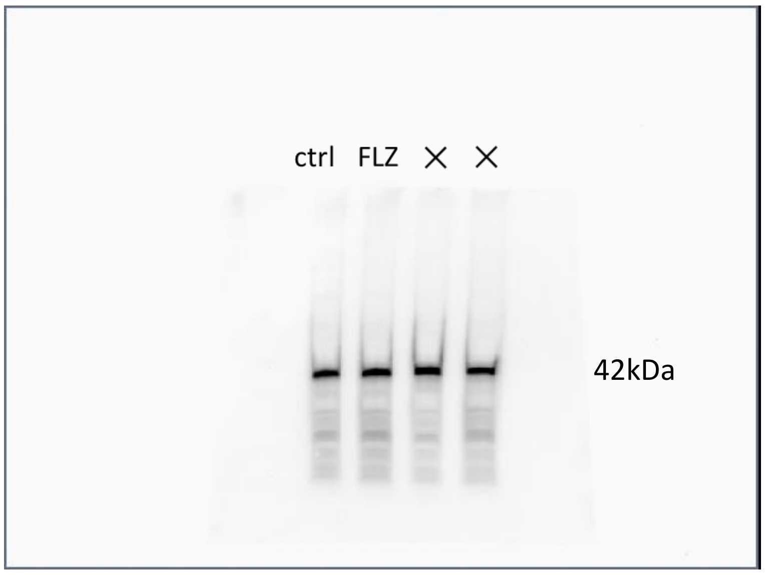

MLKL

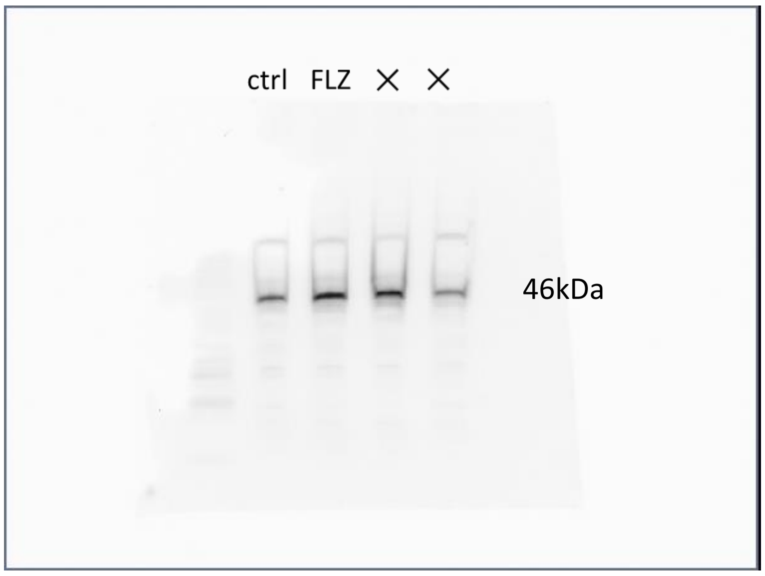

Caspase-8

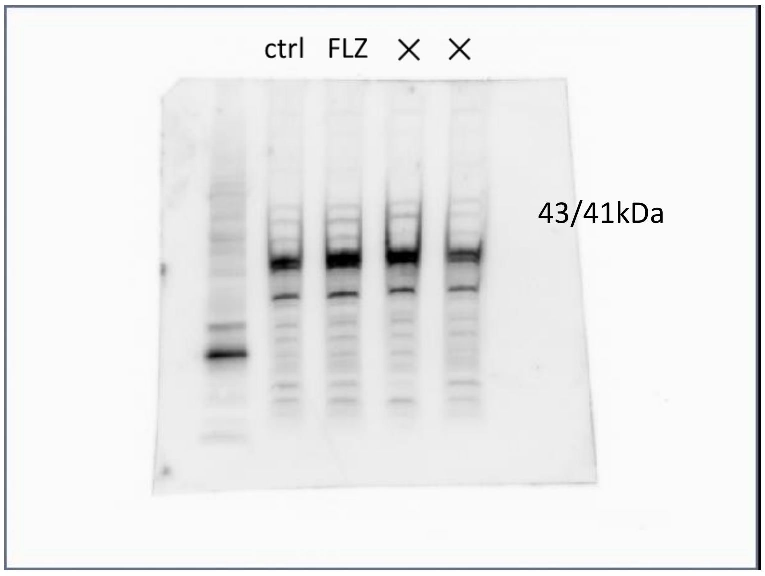

S1 Fig1e

CXCL5

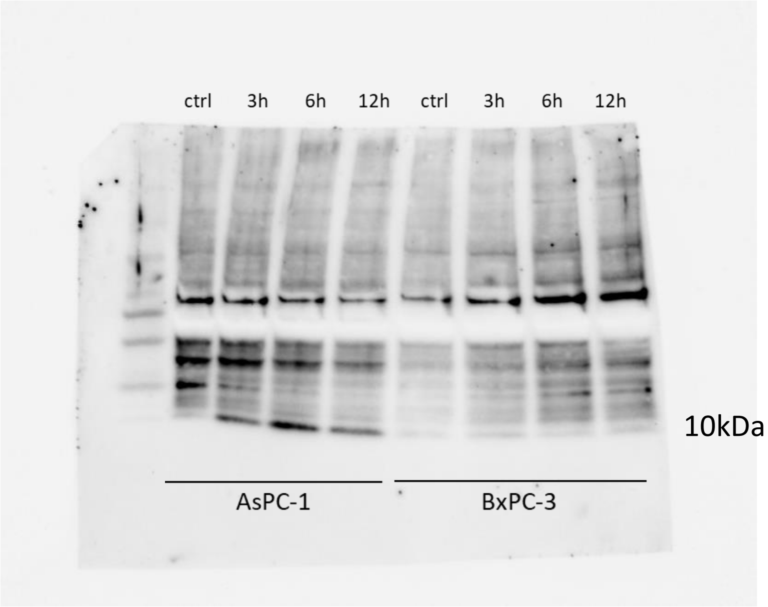

β-actin

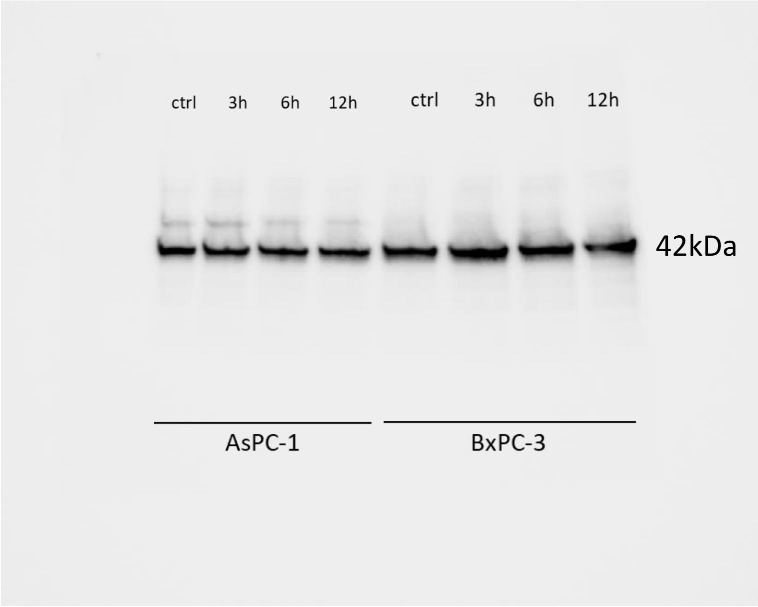

CXCR2

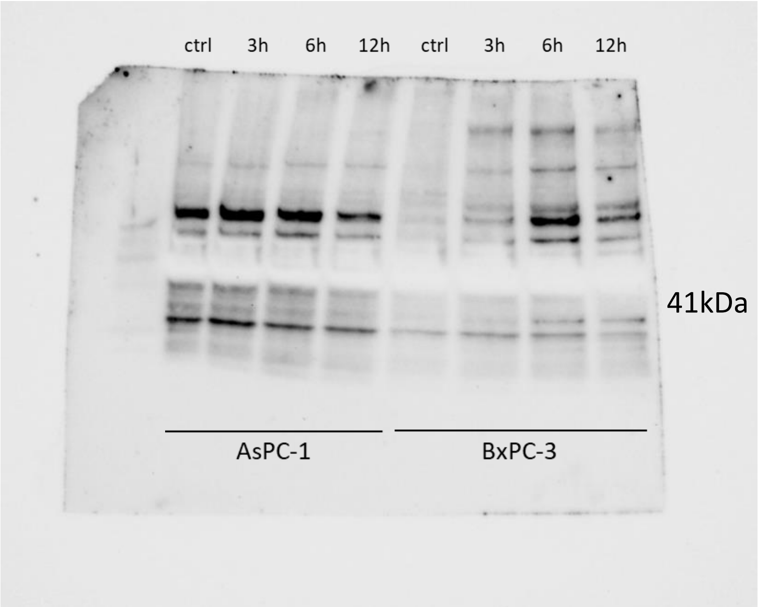

p-MLKL

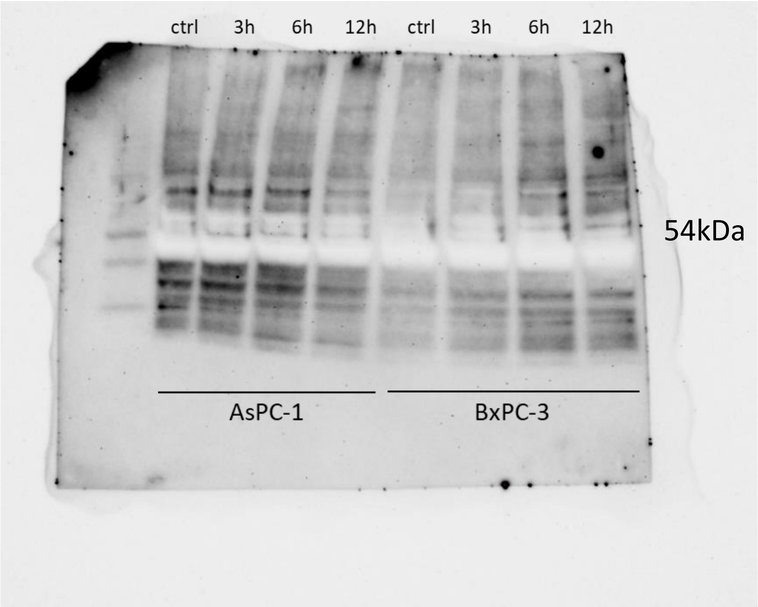

Supplement: S1 Raw images — (PDF) [file pone.0228015.s003.pdf]
